# Genomic Determinants Potentially Associated with Clinical Manifestations of Human-Pathogenic Tick-Borne Flaviviruses

**DOI:** 10.3390/ijms232113404

**Published:** 2022-11-02

**Authors:** Artem N. Bondaryuk, Nina V. Kulakova, Ulyana V. Potapova, Olga I. Belykh, Anzhelika V. Yudinceva, Yurij S. Bukin

**Affiliations:** 1 Laboratory of Natural Focal Viral Infections, Irkutsk Antiplague Research Institute of Siberia and the Far East, 664047 Irkutsk, Russia; 2 Limnological Institute, Siberian Branch of the Russian Academy of Sciences, 664033 Irkutsk, Russia; 3 Department of Biodiversity and Biological Resources, Siberian Institute of Plant Physiology and Biochemistry, Siberian Branch of the Russian Academy of Sciences, 664033 Irkutsk, Russia

**Keywords:** tick-borne flaviviruses, encephalitis, hemorrhagic fever, fixation index, cell tropism, tissue tropism

## Abstract

The tick-borne flavivirus group contains at least five species that are pathogenic to humans, three of which induce encephalitis (tick-borne encephalitis virus, louping-ill virus, Powassan virus) and another two species induce hemorrhagic fever (Omsk hemorrhagic fever virus, Kyasanur Forest disease virus). To date, the molecular mechanisms responsible for these strikingly different clinical forms are not completely understood. Using a bioinformatic approach, we performed the analysis of each amino acid (aa) position in the alignment of 323 polyprotein sequences to calculate the fixation index (*F_st_*) per site and find the regions (determinants) where sequences belonging to two designated groups were most different. Our algorithm revealed 36 potential determinants (*F_st_* ranges from 0.91 to 1.0) located in all viral proteins except a capsid protein. In an envelope (E) protein, most of the determinants were located on the virion surface regions (domains II and III) and one (absolutely specific site 457) was located in the transmembrane region. Another 100% specific determinant site (E63D) with *F_st_* = 1.0 was located in the central hydrophilic domain of the NS2b, which mediates NS3 protease activity. The NS5 protein contains the largest number of determinants (14) and two of them are absolutely specific (T226S, E290D) and are located near the RNA binding site 219 (methyltransferase domain) and the extension structure. We assume that even if not absolutely, highly specific sites, together with absolutely specific ones (*F_st_* = 1.0) can play a supporting role in cell and tissue tropism determination.

## 1. Introduction

Tick-borne flaviviruses (TBFVs) are the monophyletic group represented by 12 virus species, five of which are pathogenic to humans–the so-called ‘‘tick-borne encephalitis (TBE) serocomplex” consisting of tick-borne encephalitis virus (TBEV), louping-ill virus (LIV), Omsk hemorrhagic fever virus (OHFV), Kyasanur Forest disease virus (KFDV), and Powassan virus (POWV)) [1]. The genomes of all TBFVs comprise a single strain positive RNA encoding a polyprotein with a length from 3414 to 3416 amino acid (aa) residues cleaving into three structural and seven non-structural proteins during co-translational modification [2].

**Figure 1:**
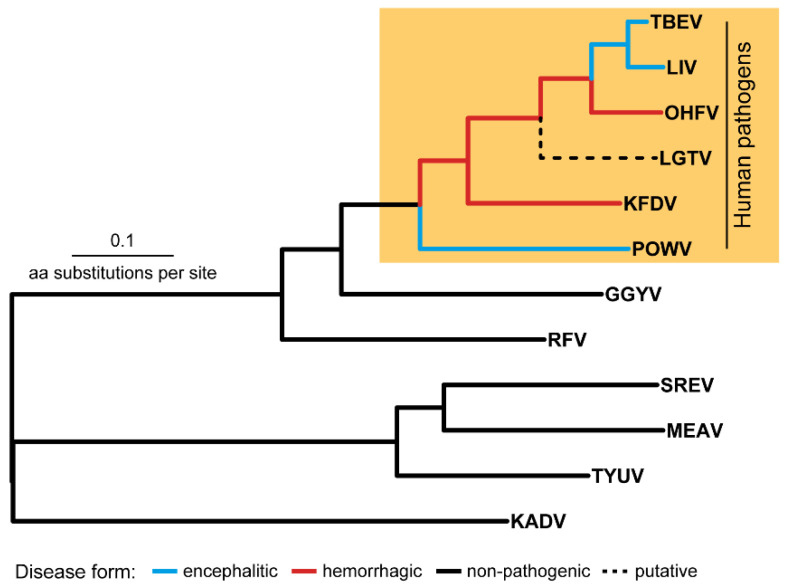
The tick-borne flavivirus species tree. Abbreviations: TBEV—tick-borne encephalitis virus, LIV—louping-ill virus, OHFV—Omsk hemorrhagic fever virus, KFDV—Kyasanur Forest disease virus KFDV, POWV—Powassan virus, GGYV—Gadgets Gully virus, RFV—Royal Farm virus, SREV—Saumarez Reef virus, MEAV—Meaban virus, TYUV—Tyuleniy virus, KADV—Kadam virus.

On the TBFV phylogenetic tree, the TBE serocomplex is the monophyletic clade (Figure 1) that also includes Langat virus (LGTV), with no registered cases of human infection (except post-vaccination encephalitis during the trials of a live attenuated LGTV-based vaccine against TBE in USSR [3]).

The members of the TBE serocomplex can be subdivided into two groups–the first group includes viruses that are able to cross the blood-brain barrier (BBB) and induce encephalitic in humans (TBEV, LIV, POWV) and the second group is comprised of pathogens causing hemorrhagic fever in humans (OHFV, KFDV) [4]. The molecular mechanisms responsible for these manifestations are not completely understood. Comprehension of these mechanisms underlying specific clinical forms can play an important role in understanding evolutionary processes in flaviviruses, drug design, the development of vaccines and other preventive measures.

The TBE serocomplex is the group of closely related viruses whose genomes accumulate mostly point aa substitutions, while indels occur less often and are similarly represented by insertions or deletions of single aa residue [5,6,7]. Therefore, differences in clinical manifestations of encephalitic (TBEV, LIV, POWV) and hemorrhagic (OHFV, KFDV) viruses are due to the mechanisms based on the point aa substitutions or indels. The problem of detection of such mutations (or determinants) is that two groups (hemorrhagic and encephalitic) do not form on the tree two independent clusters (or evolutionary lineages) which diverged in the recent past from a common ancestor (Figure 1). Flaviviruses TBEV, LIV, POWV, OHFV, KFDV are shuffled in the union cluster with basal branch of POWV (encephalitic form) followed by two hemorrhagic viruses KFDV и OHFV which in turn form an outgroup in relation to the TBEV and LIV clade. Such a shuffled topology makes it difficult to detect a common mutation responsible for different manifestations in humans. Besides, determinants in the distinct species can be defined by different aa substitutions with similar physicochemical properties that should also be counted.

At the present time, GenBank contains more than 300 complete polyprotein sequences of TBE serocomplex members (TBEV, LIV, POWV, OHFV and KFDV) each of which is presented by at least 20 molecular sequences. This sample size enables the application of population genetics methods [8] for revealing the patterns of species divergence when comparing incompletely separated (in genetic terms) groups of organisms. In our study, the incompletely separated groups are TBEV, LIV, POWV (encephalitic form) and OHFV, KFDV (hemorrhagic fever form). For this purpose, the *F_st_* criterion, which is the measure of population (intergroup) differentiation, can be employed for haploid organisms such as viruses [9]. This criterion can be modified to analyze individual positions in the polyprotein alignment of the studied groups of viruses (TBEV, LIV, POWV, OHFV, KFDV) to determine positions showing a high degree of differentiation between groups of encephalitic and hemorrhagic viruses. Such positions are candidates for determinants that define differences in the manifestation of the clinical form of viral diseases. For estimations based on aa alignments, it is possible to use substitution-rate matrices [10] (for example, the most universal JTT matrix), which indirectly allow, through the frequency of occurrence of substitutions in proteins, for a consideration of differences or similarities in their physicochemical properties.

For some structural and non-structural proteins of different flavivirus species, the spatial structures and positions of functionally significant domains have been identified [11,12,13,14]. The close relationship and polyprotein organization of all flaviviruses allow homologous modeling of the three-dimensional structures of proteins for any strain of the TBEV, LIV, POWV OHFV or KFDV group. Data on functionally significant polyprotein sites separating encephalitic and hemorrhagic viruses can help to predict their spatial localization in three-dimensional protein structures and suggest molecular mechanisms of virus-specific pathogenicity.

The current study aimed to find genetic determinants of clinical manifestations of TBE-serocomplex members (TBEV, LIV, POWV OHFV and KFDV) by analysis of complete or near complete polyprotein sequences. The study was based on a bioinformatic approach which included: (1) searching the NCBI database to form a dataset of complete polyproteins of viruses from specified groups; (2) modifying the *F_st_* criterion (the measure of intergroup differentiation) algorithm to search for molecular determinants in a polyprotein; (3) searching for polyprotein sites which are the most probable determinants of the clinical forms (encephalitis or hemorrhagic syndrome); (4) reconstruction of three-dimensional structures of proteins by homology modeling; and (5) analysis of the functional significance of the identified polyprotein sites in the three-dimensional structures of proteins.

## 2. Results

### 2.1. Molecular Determinants of Clinical Manifestations

**Figure 2:**
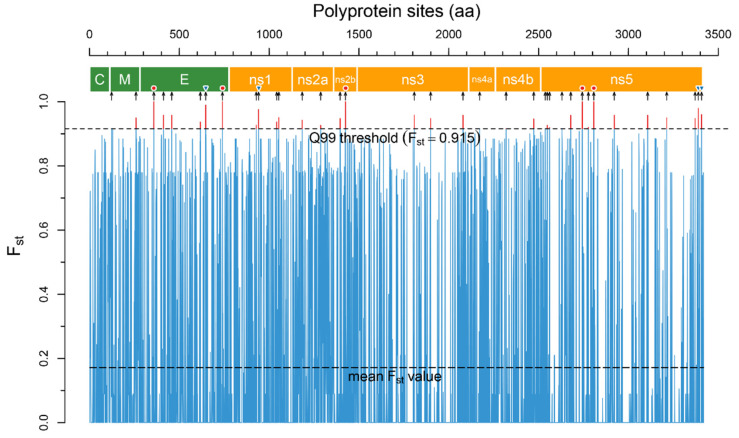
The *F_st_* plot across polyprotein sites reconstructed by the R script. *F_st_* values were calculated for two groups of aa sequences–encephalitic viruses (TBEV, LIV, POWV) and hemorrhagic viruses (OHFV, KFDV). The narrow blue trace is mean *F_st_* values for each polyprotein site, the upper dashed line is the Q99 threshold. The sites of the polyprotein with *F_st_* above the Q99 threshold (highlighted by a red color) are potential disease form determinants. The polyprotein scheme was reconstructed based on the annotation of the TBEV strain SofjinKSY (AEP25267.2): the polygons colored in green are structural proteins, those coloured orange are non-structural proteins. The black arrows under the scheme indicate 36 determinant positions in the polyprotein. Red circles above the arrows show absolutely specific positions (*F_st_* = 1.0) and blue inverted triangles show highly specific positions (*F_st_* > 0.96).

In total, the analysis revealed 1095 positions in the polyprotein with *p*-value > 0.05, 36 of which were above the accepted 99Q threshold (*F_st_* = 0.915, Figure 2) and located in all viral proteins except the capsid (C) protein (Table 1).

Five positions in E (T76A, K457R), NS2b (E63D), and NS5 (T226S, E290D) proteins have *F_st_* = 1.0 or can be considered as absolutely specific. Four positions in E (I364M), NS1 (V161M), NS5 (K872R, D890E) proteins have *F_st_* higher than 0.96 and suggested as highly specific.

Predicted positions were also checked in LGTV sequences (Table S2). All five absolutely specific positions, with the exception of one (D290 in the NS5 protein), contained specific encephalitic virus aa residues. Two of the four highly specific positions included aa residues of encephalitic viruses (NS5 protein: D890), one–an aa residue of hemorrhagic viruses (E protein: M364), one position contained a unique for LGTV aa residue (NS1 protein: I161) and the last one comprised both encephalitic (NS5: K872) and hemorrhagic (NS5: R872) markers in different LGTV sequences.

The sites with *F_st_* values above the Q99 threshold were extracted to perform the verificative phylogenetic reconstruction.

### 2.2. Phylogenetic Proof

**Figure 3:**
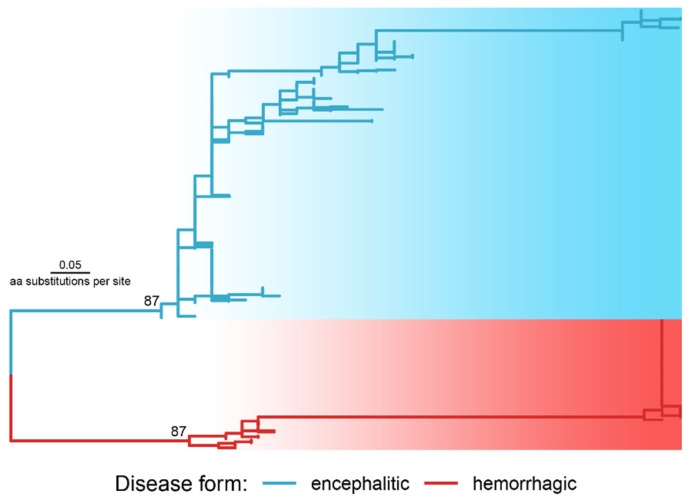
The phylogenetic tree reconstructed with the polyprotein regions selected as molecular determinants of disease forms (a newick tree file is available at https://doi.org/10.6084/m9.figshare.21154495, accessed on 1 October 2022). The tree was rooted in a midpoint of the two longest tips. The numbers at the nodes are ultrafast bootstrap values.

Phylogenetic analysis using 36 preliminary extracted candidate positions with *F_st_* above the Q99 threshold inferred the explicit division of sequences into two clusters according to disease forms (Figure 3).

The obtained subdivision verified the accepted threshold. At the lower threshold values, sequences from viruses inducing different clinical forms are shuffled on the tree (Figure S1) taking the topology of the complete polyprotein tree (Figure 1).

### 2.3. Reconstruction and Visualisation of Atomic Structures

Three-dimensional structures for six out of ten viral proteins corresponding to the parts of the TBEV strain SofjinKSY polyprotein and carrying sites which are specific for the clinical forms were reconstructed using the SWISS-MODEL algorithm (Table 2). The template sequences of three-dimensional structures of the reconstructed structural preM, M, E and non-structural NS1, NS3, NS5 proteins from the Protein Data Bank (PDB) had a similarity with those of SofjinKSY, ranging from 42.12% to 96.88%. For the proteins preM, M and E, the best template sequences were structural proteins of the European TBEV strain Kuutsalo-14 (PDB id: 7z51). For the non-structural proteins NS1, NS3, NS5 of the strain SofjinKSY, the best templates were three-dimensional structures of corresponding proteins of the viruses Zika, Dengue and Japanese encephalitis.

**Figure 4:**
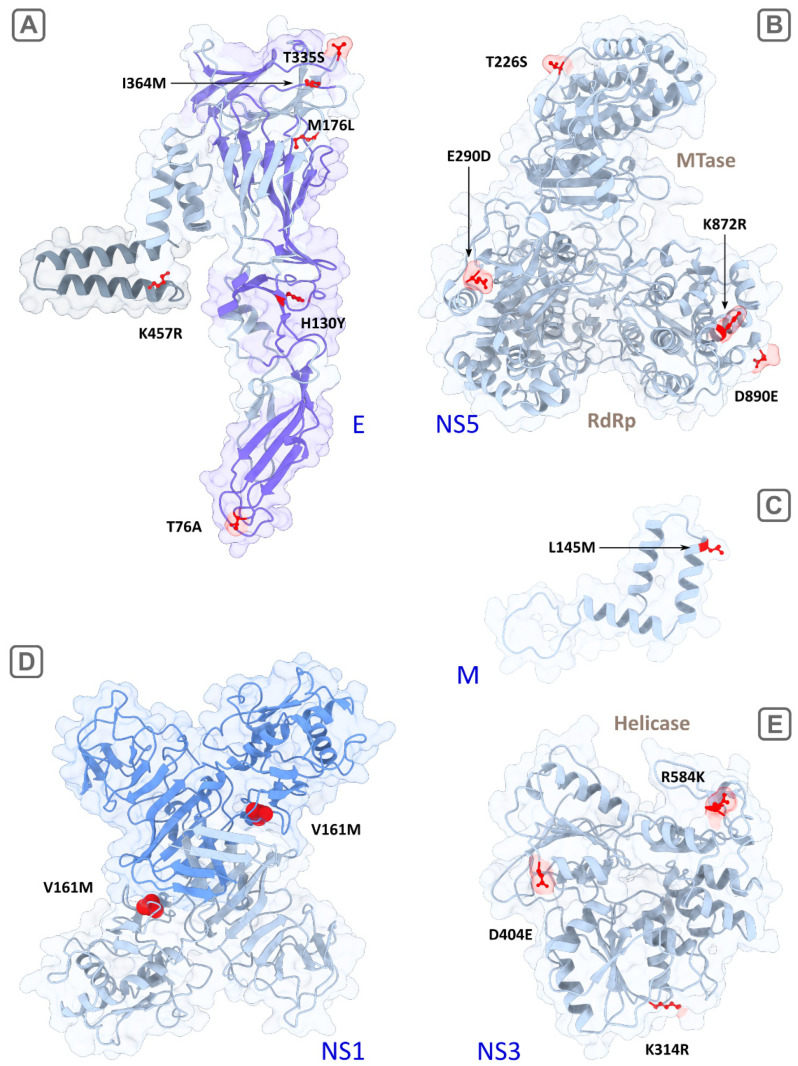
Visualization of reconstructed 3D structures of TBEV proteins, with the strain SofjinKSY used as a template (AEP25267.2). The suggested determinants of encephalitic and hemorrhagic clinical forms are colored in red. (**A**) the E protein monomer, the transmembrane region is highlighted in grey, the surface region (front sheet) is highlighted in purple; (**B**) the full-length NS5 protein including the RNA-dependent RNA polymerase (RdRp) and methyltransferase (MTase) domains; (**C**) the fragment of the M protein (94–167 aa); (**D**) the dimer of NS1, with the monomers highlighted in grey and blue; and (**E**)—the helicase domain of NS3. Ribbons are highlighted in light gray, molecule surfaces are transparent.

Visualized three-dimensional structures of the proteins in strain SofjinKSY are shown in Figure 4. All studied virus proteins have similar three-dimensional structures due to their close relationship, structural and functional similarities.

## 3. Discussion

Our algorithm identified 36 determinants of the clinical forms in all proteins, except for the capsid C protein. In the previous studies [4], it was found that hemorrhagic viruses share sites located in in the envelope E protein (position 76 in OHFV Lin, et al. (2003) [15]) and two in the NS3 protein (558 and 585 in OHFV corresponding to 557 and 584 in TBEV, strain SofjinKSY, AEP25267.2). In our study, the position 557/558 (OHFV/TBEV) with mean *F_st_* = 0.87 did not exceed the 99Q threshold and was therefore not included in the following analysis.

We were unable to reconstruct the structures of NS2a, NS2b, NS4a, NS4b proteins due to the absence of homologues in PDB. In addition, they did not contain absolutely specific sites (except highly specific one in NS2b). Therefore, we restricted our discussion to M, E, NS1, NS2b, NS3, NS5 proteins whose roles in virus pathogenesis are more studied.

### 3.1. Predicted Determinants in the Reconstructed Structures

#### 3.1.1. M Protein

The mature M protein is a part of the viral membrane and initially includes precursor part (pr) which splits from M in the Golgi complex of infected cells [16]. The prM protein forms a tight, heterodimeric complex with the E protein and plays an important role in virus assembly [17]. Two potential determinants were detected in the M protein–the low-specific substitution (K9R, *F_st_* = 0.91) in the N-terminus of the protein and the another more specific one (L145M, *F_st_* = 0.95) in the C-terminal region consisting of two potential membrane-spanning domains [2] (Figure 4C). K9R in the M protein is located in the contact region with the envelope protein E during the maturation phase before the cleavage of preM by proteases [18]. Thus, changes in this position of the preM protein can affect the intracellular processes of virus persistence and maturation of viral particles. L145M is located in the region of the hydrophobic alpha helix at the site of its penetration into the inner part of the viral particle through the lipid membrane. Together with the envelope protein E, the M protein is responsible for the transformation of the viral membrane during the penetration into the host-cell and the release of viral RNA [19].

#### 3.1.2. E Protein

The E protein is an antiparallel dimer that is oriented horizontally to the viral membrane [20], wherein, each of a monomer has three domain structures (domains I, II, III). A comparison of atomic structures of the E protein in a number of flaviviruses (e.g., Japanese encephalitis virus, West Nile virus, yellow fever virus, Zika virus) revealed the same common protein architecture that enables us to visualize and compare molecular determinants of related TBFVs using the TBEV E protein structure (PDB id: 7z51).

Our algorithm detected six candidate sites (76, 130, 176, 335, 364, 457), four of which are located on the ‘front sheet’ of the E protein (virion surface) [20], one on the ‘back sheet’ and the last in the transmembrane region (Table 1; Figure 4A). Predominant localization on the surface and in the transmembrane domain indicated the potential functional significance of these sites. In particular, detected by the algorithm and described previously [4], the substitution T76A with a maximum *F_st_* of 1.0 value is located in the bc loop of the domain II (surface) and likely to interact with the fusion peptide (cd loop) in the same domain [20]. Alanine has hydrophobic side chain and is unable to form hydrogen bonds, wherein threonine is hydrophilic and is able to form one hydrogen bond. So, a T→A aa substitution can theoretically change the functional properties of the protein (particularly, cell tropism). The mutation H130Y replaces hydrophilic aa (H) with hydrophobic one (Y), wherein side-chain volume of Y (203) is bigger than of H (167). Other two aa substitutions (T335S, I364M) lying on the front sheet of the E protein do not change protein physical properties significantly, but still can influence the process of fusion of viral and cellular membranes. Another aspect which can crucially influence tissue tropism is attachment factors on a cell surface serving as receptors or co-receptors for virus binding. Some of the most studied attachment factors are glycosaminoglycans (GAGs), dendritic cell-specific intercellular adhesion molecule-3-grabbing non-integrin (DC-SIGNs) and its paralog–DC-SIGN-related molecules (DC-SIGNR) [21,22]. GAGs and DC-SIGNRs, in particular, are expressed on microvascular endothelial cells which can affect neuroinvasiveness or potentially induce hemorrhagic syndrome. A GAG molecule is a negatively charged polysaccharide, well known as an attenuation factor of flaviviruses [23]. GAG-binding sites are mainly located in the domain III of the E protein and they continue to be discovered [24]. Moreover, there is a report on a putative GAG-binding site (E138 in Japanese encephalitis virus) in the domain I [25]. It was demonstrated that high affinity to GAGs mediated by accumulation of positively charged residues on the E protein surface leads to decreasing neuroinvasiveness in a mouse model [26]. The mechanism of attenuation of flaviviruses is thought to be related to an inability of the strains with high affinity to GAGs to produce enough level of viremia of sufficient magnitude and/or duration required for brain invasion [27]. In our study, predicted determinants in the domain III do not change a charge of aa residues and may only have an effect on the spatial location and accessibility of GAG-binding sites. We also speculated that the determinants located in the other domains (for example, the substitution of positively charged histidine by uncharged hydrophobic tyrosine in the position 130) are potential GAG-binding sites. It also known that N-glycosylated surface proteins of the virus can interact through their glycans with C-type lectins such as DC-SIGN [23]. Determinants predicted in this study are not glycosylation sites of TBFVs (67 and 154) [28]. Presumably, these determinants can only have a spatial effect on DC-SIGN binding by viral glycans. As a whole, it was noted that, even applying informative site-directed mutagenesis, it is difficult to find a relationship between the virus and specific cell receptors [29].

The one additional mutation K457R in the E protein with absolute specificity (*F_st_* = 1.0) is located in the transmembrane region. It replaces two positive-charged aa residues with similar physicochemical properties but lysine is capable of forming two hydrogen bonds and arginine is capable of forming four bonds side chains. The anchored into cellular and viral membranes transmembrane domains in the proteins E and M play a crucial role in maturation of flavivirus envelope. Their anchor function is necessary to isolate a fraction of a cellular membrane that becomes part of the viral envelope [17,30] (for more detailed scheme of virus entry see Hu, et al. (2021) [29]). We speculate that mutations in the transmembrane region (such as L145M in the M protein and K457R in the E protein) which distinguish two groups can affect the zippering reaction and change the cell and tissue tropism of viruses [19].

In general, mutations located on the virus surface can change the degree of the binding affinity of viruses to receptors on the host-cell surface (directly or indirectly) or influence virus entry at the stage of membrane fusion, which can affect the tropism of viruses to various tissues or virus entry activity.

#### 3.1.3. NS1 Protein

NS1 interacts with various host proteins to facilitate viral replication, translation, and virion production [16,31]. Also, in the form of a hexamer, NS1 is secreted in the blood, where it plays a role in immune system evasion [32]. Four detected determinants are located in the second “wind” domain (R148K, V161M) and in the C-terminal central β-ladder domain (S262A, I274L) (Figure 4D). The most specific substitution was V161M (*F_st_* = 0.976); however, the physicochemical properties of valine and methionine are similar. The substitution S262A changes the polar uncharged serine (with one potential hydrogen bond) to the hydrophobic alanine (zero hydrogen bond) that likely affects NS1 functioning. Besides, site 262 is located in the region of antibody binding [33].

#### 3.1.4. NS2b Protein

NS2b is a crucial co-factor for protease activity of the NS3 protein which, in turn, is a polyfunctional protein and acts as a serine protease, helicase, and RNA nucleoside triphosphatase. One absolutely specific mutation (E63D, mean *F_st_* = 1.0 with the exception of one sequence with an alternative allele K in the encephalitic group) lies in the central hydrophilic domain of the NS2b that mediates NS2b activity [34].

#### 3.1.5. NS3 Protein

All determinants detected in NS3 (K314R, D404E, R584K) are located in the C-terminus (helicase domain) and two of them (314, 404) are in conservative motives (III and V, respectively; Figure 4E). They are not absolutely specific, but side chains of K and R can form a different number of hydrogen bonds (2 and 4, respectively).

#### 3.1.6. NS5 Protein

NS5 is the longest viral protein component within the replicative complex of TBFVs. In NS5, 14 substitutions with different specificities (*F_st_* ranges from 0.916 to 1.0) were detected in our analysis as potential determinants of the clinical forms. Of these, the H696P (*F_st_* = 0.95) substitution, with positive charged (+1) histidine replaced by uncharged proline might be the most important.

A position 696 is in the inter-domain interface involved in binding the STAT2 protein [35]. Inhibition of the STAT2 protein blocks innate immunity [36].

Other detected substitutions are spatially located near active sites of methyltransferase (MT) and RNA-depended RNA polymerase (RdRp) domains (Figure 4B). Two absolutely specific substitutions, T226S and E290D, are located in MT and the extension structure (slate) connecting MT with RdRp via the linker. The first mutation (T226S) lies near the RNA binding site 219–the part of the MT catalytic tetrad KDKE crucial for methylation of viral RNA, and, therefore, the substitution in this site likely affects the activity of MT. The role of extension structure is not completely understood, it was supposed that it may play auxiliary roles to RdRp during RNA synthesis de novo [13]. Thus, the functional significance of the E290D substitution is unclear.

### 3.2. Possible Influence of Vector/Host Specificity

In our study, we subdivided our dataset by clinical form. However, the results obtained can be biased by other signals in the data. It is known that arboviruses, including the family *Flaviviridae*, are under selective pressure in vertebrate and invertebrate hosts [37]. The viruses of the *Flavivirus* genus, for example, demonstrate a clear correlation between phylogenetic relationships and virus–vector interactions [7] when tick and mosquito viruses form independent monophyletic clusters on the tree. Even so, at a lower level, the TBFV cluster did not exhibit host-specific associations (Table 3). Within the hemorrhagic viruses, invertebrate hosts (or vectors) differ at the family level, whereas the range of vertebrate hosts is much wider and represented by small mammals, primates, bats, birds, etc. Vectors of encephalitic viruses are mainly *Ixodes spp.* ticks, but it was reported that *Dermacentor reticulatus* also might play a relevant role as a TBEV nature reservoir [38]. Moreover, TBEV was detected in pools of *Haemaphysalis punctata* [39] and other *Haemaphysalis* spp. [40]. There is a report on the isolation of POWV from *H. longicornis* [41]. Concerning vertebrate hosts, numerous species of mammals and birds are TBEV reservoirs [42] (p. 57). POWV-positive samples were collected from white-footed mice, deer and squirrels [43]. LIV, in turn, has the unique structure of natural foci where the virus is transmitted between red grouse, sheep and mountain hares [44]. So, we did not find *F_st_* associations with vector or host specificity.

### 3.3. Absolutely Specific Determinants Indicate LGTV Neurovirulence

Analysis of LGTV sequences using predicted determinants showed that four of five absolutely specific positions comprised aa residues of encephalitic viruses. Although the highly specific positions do not provide unanimous conclusions on eventual LGTV disease form (Table S2), we suppose that absolutely specific markers point to the LGTV neuroinvasiveness/neurotropism. This speculation is supported by the fact that during the trials of live attenuated LGTV-based vaccine against TBE in USSR it was reported on high frequency of encephalitis (1:18,570) [45]. Some of LGTV strains also exhibited neurovirulence in mice and monkeys [46]. Thus, at least four of the five absolutely specific sites predicted in our study are presumed to be as relatively reliable encephalitic markers.

### 3.4. The Role of Point Amino Acid Substitutions and Potential for Further Molecular Dynamics Simulations and Animal Testing

There are several bioinformatic predictions of hot spots in genomes which affect different viral properties including cell and tissue tropism [47,48,49,50]. Some of them are proven in practice. For example, a recent study showed that a predicted single T403R mutation increases binding of S protein of Bat coronavirus RaTG13 (a close relative of SARS-CoV-2) to human ACE2 cell receptor [51].

In concordance with the previous study [4], we found no aa motives in polyproteins affecting TBFV clinical manifestations in humans. Only point aa substitutions were detected. In fact, it was shown that one or a few aa substitutions are sufficient to change virus properties dramatically. This is especially well illustrated by the example of the S protein of the SARS-CoV-2. So, the replacement G614D alone in the SARS-CoV-2 spike protein enhances the virus infectivity [52]. The substitution L452R enables virus to evade cellular immunity [53].

The determinants found in our study can also be tested by molecular dynamics (MD) simulations or by site-directed mutagenesis with animal testing. The MD method is intended for analyzing the movements of atoms in a molecular system, which are described by classical Newton’s equations of motion. The MD simulation assumes the free interaction of atoms during a certain period of time, which is reflected in the dynamic “evolution” of the system. The search for local and global minimum energy of a molecular system allows one to evaluate the stability of ensemble conformations for a certain protein. By comparing protein sequences with different point aa mutations, we can find their contribution to the stability and properties of a molecular system. In particular, MD allows us to calculate the interaction dynamics of various mutant proteins (for example, different variants of the E protein) in interaction complexes with cell receptors and determine their ability to penetrate cells of various tissues. MD models show a temporal stability of protein complexes of different viral variants and cellular proteins which are formed during virus entry into cells thus determining tropism for various host tissues. With the correct determinant prediction, it will be possible to change virus properties (cell tropism) and, as a consequence, their clinical manifestations.

## 4. Materials and Methods

### 4.1. Protein Sequences

The 323 polyprotein sequences of TBE-serocomplex members with mean length of 3414 aa were downloaded for the analysis from GenBank in February 2022 (Table 3):

**Table 3:** Summary of sequences used in the analysis.

Virus	Number of Sequences	Disease Form ^1^	Invertebrate Hosts	Vertebrate Hosts
KFDV	54	Hem	*Haemaphysalis spinigera* [1]	Monkeys, small mammals, bats [54]
AHFV ^2^	21	Hem	*Ornithodoros savignyi, Hyalomma dromedarii*	Sheep [55]
OHFV	21	Hem	*Dermacentor reticulatus* [56], *Ixodes persulcatus* [57]	*Microtus gregalis, Ondatra zibethicus* [58,59]
POWV	23	Enc	*I. cookei, I. marxi, I. scapularis* [43], *H. longicornis* [41]	*Peromyscus leucopus, Odocoileus virginianus, Tamiasciurus hudsonicus* [43]
LIV	26	Enc	*I. ricinus*	*Lagopus lagopus scotica*, sheep [44]
TBEV ^3^	178	Enc	*Ixodes spp., D. reticulatus* [38], *H.* spp. [39,40]	numerous mammal and bird species [42] (p. 57)

^1^ Hem–hemorrhagic form, Enc–encephalitic form; ^2^ Alkhumra hemorrhagic fever virus (AHFV) is subtype of KFDV; ^3^ The TBEV group includes all TBEV subtypes and single lineages.

The sequences in the data set were labeled as hemorrhagic viruses (96 sequences) and encephalitic viruses (227 sequences), filtered by stop codons and aligned with MAFFT v.7.475 [60].

LGTV were not included in the alignment as it is not associated with human disease under natural conditions. Instead, we analyzed three available LGTV polyprotein sequences in the last stage of this study following the determinants predicted by our search algorithm.

### 4.2. Search Algorithm for Genetic Determinants

An original algorithm in the R programming language was developed to identify sites in virus polyprotein which differentiate viruses by their clinical form (hemorrhagic syndrome or encephalitis) in human. The algorithm consists of the following steps:

1. Obtaining an aa substitution-rate matrix based on the universal model JTT [61], normalized in the range from 0 to the maximum value. In the original JTT model, substitution weights are changing from −5 (most common substitutions) to 5 (most rare substitutions). Substitutions with a weight of −5 were assigned as 1, substitutions with a weight of 5 were assigned as 10, and rest was converted according to this range of values. Gaps (indels) with the highest weight 11 were additionally added to the weight matrix; Applying of JTT matrix of substitution weights allowed us to estimate differences in substitutions` significance for adaptive transformations due to different physical-chemical properties of residues (mutations which led to significant changes in aa properties is rare).
2. For each position in the alignment, a matrix of pairwise evolutionary distances was calculated. If the aa residues in the two compared sequences at a given position matched, then the pairwise distance was 0; if the aa residues did not match, the distance was taken as a weight of the aa substitution from the transformed JTT matrix. Based on the matrix of pairwise evolutionary distances for each position, the average intragroup *H_w_* and intergroup *H_b_* distances (for the “encephalitic” and “hemorrhagic” groups) were calculated. Based on *H_w_* и *H_b_*, the *F_st_* criterion (fixation index) [9] was calculated, showing the degree of intergroup differentiation according to Formula (1):
  (1) $$F_{st}=1-\frac{H_{w}}{H_{b}}$$
  Values *F_st_* range from 0 to 1, values close to 0 indicate the absence of intergroup subdivision, values close to 1-high subdivision. If *H_w_* ≥ *H_b_* or there were no substitutions in a particular position, then *F_st_* was assigned 0.

3. A bootstrap analysis was used to verify estimated *F_st_*, according to the following scheme: from each group (“encephalitic” and “hemorrhagic”) of polyprotein sequences, a replica was selected from 96 random sequences with a return (according to the smallest sample size of viruses that cause hemorrhagic fevers). For each position of each replica, *F_st_* was calculated. The procedure was repeated 2000 times. Thus, 2000 *F_st_* values were obtained for each aligned position. The probability of the null hypothesis-no differentiation was calculated using the formula:
  (2) P=n/2000
  where *P*–the probability of the null hypothesis (*p*-value), *n*–the number of replicas with *F_st_* = 0. If *p* > 0.05 then *F_st_* value was replaced by 0 (no differentiation). For further analysis, the average value of *F_st_* from 2000 bootstrap replicas was taken for each position.

4. Finally, *F_st_* values for all positions were ranged in the ascending order from 0 to 1 with a step of 0.01. The quantile (Q) of the largest *F_st_* values (excluding Q0) was selected with the formation of new datasets (subsets) from the alignment, with the highest *F_st_* values. From 100 obtained subsets, each next subset (ascending) contained fewer alignment positions, but with higher *F_st_* values and increasing mean differentiation between groups (encephalitis and hemorrhagic). For each of 100 subsets, a phylogenetic tree was constructed using the UPGMA method using the JTT distance matrix. The structure of each tree was analyzed visually. The subset with the minimum quantile of the ranked *F_st_* was selected, in which the tree was divided into two monophyletic clusters, one of which included only species that cause hemorrhagic fevers, and the other cluster included only encephalitis.
5. Thus, selected subset of data was considered the candidate dataset to search determinants of different clinical manifestations of virus manifestation. For the statistical assessment of the tree topology, we performed additional phylogenetic analysis in IQTREE v.1.6.12 [62] with the ultrafast bootstrap support [63] and model selection using ModelFinder [64] implemented in IQTREE.

To implement the algorithm in R, additional packages were used: seqinr [65]–to download and edit protein sequences; bios2mds [66]–to make the initial dataset for JTT weight matrix of aa substitutions; phangorn [67]–to reconstruct evolutional trees using UPGMA and the JTT model; ggtree [68]–to visualize phylogenetic trees. A script in R with the implemented algorithm and the initial alignment of the complete polyprotein sequences are available at the link-https://doi.org/10.6084/m9.figshare.21218594 (accessed on 1 October 2022).

### 4.3. Reconstruction, Visualization, and Analysis of 3D Models of Protein Molecules

For the reconstruction of three-dimensional protein structures, we chose the TBEV polyprotein of the strain SofjinKSY (NCBI accession number: AEP25267.2) as a template. The viral proteins (M, E, NS1, NS2a, NS2b, NS3, NS4a, NS4b, NS5) containing candidate positions separating hemorrhagic fevers and encephalitis were determine from this polyprotein using NCBI annotation. The reconstruction of three-dimensional protein structures was carried out using the SWISS-MODEL online server (https://swissmodel.expasy.org/interactive, accessed on 1 October 2022) [69]. From a set of reconstructions, a model for with the highest identity with template sequence was selected. If an identity with template sequence exceeded 30% the structure was considered sufficient for the analysis. The reconstructed protein structures were saved in pdb format for further manipulations.

Three-dimensional structures of proteins were visualized using UCSF ChimeraX [70]. The spatial positions of aa residues of candidate determinants, separating hemorrhagic fevers and encephalitis, were marked on three-dimensional structures.

Comparing physicochemical properties of aa residues was performed using APDbase [71].

## 5. Conclusions

We believe that, despite the fact that not all detected positions are absolutely specific, their locations and resulting changes of physicochemical properties in conjunction with other absolutely specific positions (epistasis) play roles of determinants of clinical manifestations and affect cell and tissue tropism of viruses. In particular, this applies to:

- the E protein where the most of determinants lie on the front sheet of the virion surface and one–in the transmembrane region. These sites take part in virus budding and membrane fusion which in total can affect cell tropism;
- non-structural proteins NS1, NS3 and NS5 which provide intracellular persistence of viruses [18] while mutations in them facilitate changes in a tropism to various tissues at the intracellular level and immune response;
- the NS5 protein with determinants located on the inter domain interface and at the regions near active sites.

Our hypothesis can be confirmed by experimental data (site-directed mutagenesis and studies involving animals) or by molecular dynamics analysis. The latter is our main goal in the near future.

## Figures and Tables

**Table 1:** The molecular determinants of clinical manifestation.

Protein	Position ^1^	Residue ^2^		Mean *F_st_*	Domain	Note
		Enc_(enc/hem,%)_	Hem_(hem/enc,%)_			
M	9	K_(87/22)_	R_(78/13)_	0.916	N-terminus	
	145	L_(98/0)_	M_(98/2)_	0.950	transmembrane region	
E	**76** ^3^	T_(100/0)_	A_(100/0)_	1.000	bc loop, domain II	front sheet ^4^
	130	H_(88/16)_	Y_(84/12)_	0.958	e strand, domain II	front sheet
	176	M_(78/22)_	L_(78/22)_	0.958	G_0_H_0_ loop, domain I	back sheet
	335	T_(77/22)_	S_(78/22)_	0.937	BC_x_ loop, domain III	front sheet
	364	I_(100/1)_	M_(99/0)_	0.989	D_x_E loop, domain III	front sheet
	**457**	K_(100/0)_	R_(100/0)_	1.000	transmembrane region	
NS1	148	R_(92/0)_	K_(100/8)_	0.926	“wing” domain	
	161	V_(99/0)_	M_(99/0)_	0.976	“wing” domain	
	262	S_(84/22)_	A_(78/16)_	0.937	C-terminal domain	antibody binding region
	274	I_(80/22)_	L_(78/19)_	0.950	C-terminal domain	
NS2a	52	R_(62/0)_	T_(100/0)_	0.943		
	155	L_(90/17)_	Y_(78/0)_	0.926		
NS2b	33	V_(89/8)_	A_(92/0)_	0.947		
	**63**	E_(99.4/0)_	D_(100/0)_	0.99		
NS3	314	K_(89/15)_	R_(85/11)_	0.958	helicase domain	motif III
	404	D_(77/22)_	E_(78/22)_	0.947	helicase domain	motif V
	584	R_(96/8)_	K_(92/4)_	0.958	helicase domain	
NS4a	56	M_(87/22)_	V_(78/13)_	0.916		
NS4b	54	I_(86/22)_	M_(78/14)_	0.916		
	208	L_(100/0)_	V_(80/0)_	0.947		
NS5	20	K_(68/24)_	R_(76/32)_	0.916	MT domain	near the GTO binding site
	31	I_(90/18)_	V_(82/10)_	0.926	MT domain	near the GTO binding site
	44	R_(96/7)_	K_(93/3)_	0.919	MT domain	
	113	K_(84/7)_	R_(93/16)_	0.916	MT domain	near the active MT site
	162	K_(75/22)_	R_(78/25)_	0.958	MT domain	near the active MT site
	**226**	T_(100/0)_	S_(100/0)_	1.000	MT domain	near the RNA binding site 219
	260	V_(82/22)_	T_(78/14)_	0.920	MT domain	
	**290**	E_(99.6/0)_	D_(100/0.4)_	1.000	extension structure	
	404	K_(78/22)_	R_(78/22)_	0.958	fingers subdomain	
	590	I_(80/22)_	V_(78/20)_	0.958	palm subdomain	
	696	H_(78/22)_	P_(78/22)_	0.950	inter-domain interface	binding the STAT2 protein
	854	K_(96/0)_	R_(100/4)_	0.947	thumb subdomains	
	872	K_(96/4)_	R_(96/4)_	0.979	thumb subdomains	
	890	D_(99/0)_	E_(100/0)_	0.960	thumb subdomains	

^1^ The protein positions are given according to the TBEV strain SofinKSY (AEP25267.2); ^2^ The proportion (%) of a dominant amino acid (aa) residue in a determinant site for encephalitic (Enc) and hemorrhagic (Hem) viruses. In parenthesis, proportions are given via “/” for the target group in comparison with the opposite one to illustrate homoplasy. See the full list of site polymorphism at Table S1 and the consolidated alignment (https://doi.org/10.6084/m9.figshare.21154489, accessed on 1 October 2022); ^3^ The sites with *F_st_* = 1.0 are bolded; ^4^ Spatial disposition relative to the virion surface.

**Table 2:** Information on Reconstruction of 3D structures of TBEV proteins, the template strain SofjinKSY (AEP25267.2).

Protein in the Strain SofjinKSY	Closely Related Atomic Structure from PDB	Similarity Degree between SofjinKSY and PDB Structure (%)	Structural Region Length (aa)	Coordinates of a Structural Region in SofjinKSY	Coordinates of a Structural Region in a Polyprotein
preM	7qrf ^1^	96.88	79	6–84	118–196
M	7z51 ^2^	88.00	74	94–167	206–279
E	7z51	95.36	494	1–494	281–774
NS1	5gs6 ^3^	42.12	351	2–352	778–1128
NS2a	- ^4^	-	-	-	-
NS2b	-	-	-	-	-
NS3	2whx ^5^	45.75	599	23–621	1512–2110
NS4a	-	-	-	-	-
NS4b	-	-	-	-	-
NS5	4k6m ^6^	56.58	887	5–891	2516–3402

^1^ Structure of the dimeric complex between a precursor membrane ectodomain (prM) and an envelope protein ectodomain (E) of TBEV; ^2^ The small membrane protein (M) in a complex with the envelope protein (E) of TBEV; ^3^ The NS1 protein of Zika virus; ^4^ Dashes mean inability to reconstruct a 3D structure due to the absence of homologues in PDB; ^5^ A second conformation of the NS3 protease-helicase from dengue virus; ^6^ Crystal structure of the full-length Japanese encephalitis virus NS5 protein.

## Data Availability

All data used and obtained during this study can be found at link: https://figshare.com/projects/Genomic_determinants_of_clinical_manifestations_of_TBFVs_which_are_pathogenic_to_humans/149266 (accessed on 1 October 2022).

## Supplementary Materials

The following supporting information can be downloaded at: https://www.mdpi.com/article/10.3390/ijms232113404/s1.
