# Supplementary material for: Genomic Determinants Potentially Associated with Clinical Manifestations of Human-Pathogenic Tick-Borne Flaviviruses
Source: Int J Mol Sci. 2022 Nov 2;23(21):13404. doi: 10.3390/ijms232113404 (PMC9658301; doi:10.3390/ijms232113404)
Supplement: Supplementary file 1 [file ijms-23-13404-s001.zip › Table S2.pdf]

**Protein**	**Position**	**Mean F_{st}**	**Enc residue**	**Hem residue**	LGTV residue	Number of LGTV sequences	Highly specific	Absolutely specific
M	9	0.916	K$_{(87/22)}$	R$_{(78/13)}$	R	3		
	145	0.950	L$_{(98/0)}$	M$_{(98/2)}$	L	3		
E	**76***	1.000	T$_{(100/0)}$	A$_{(100/0)}$	**T**	3		
	130	0.958	H$_{(88/16)}$	Y$_{(84/12)}$	Y	3		
	176	0.958	M$_{(78/22)}$	L$_{(78/22)}$	L	3		
	335	0.937	T$_{(77/22)}$	S$_{(78/22)}$	T	3		
	364	0.989	I$_{(100/1)}$	M$_{(99/0)}$	M	3		
	**457**	1.000	K$_{(100/0)}$	R$_{(100/0)}$	**K**	3		
NS1	148	0.926	R$_{(92/0)}$	K$_{(100/8)}$	R	3		
	161	0.976	V$_{(99/0)}$	M$_{(99/0)}$	I	3		
	262	0.937	S$_{(84/22)}$	A$_{(78/16)}$	A	3		
	274	0.950	I$_{(80/22)}$	L$_{(78/19)}$	V	3		
NS2a	52	0.943	R$_{(62/0)}$	T$_{(100/0)}$	S	3		
	155	0.926	L$_{(90/17)}$	Y$_{(78/0)}$	L	3		
NS2b	33	0.947	V$_{(89/8)}$	A$_{(92/0)}$	L	3		
	**63**	0.99	E$_{(99.4/0)}$	D$_{(100/0)}$	**E**	3		
NS3	314	0.958	K$_{(89/15)}$	R$_{(85/11)}$	R	3		
	404	0.947	D$_{(77/22)}$	E$_{(78/22)}$	K/R	1/2		
	584	0.958	R$_{(96/8)}$	K$_{(92/4)}$	R	3		
NS4a	56	0.916	M$_{(87/22)}$	V$_{(78/13)}$	F/L	1/2		
NS4b	54	0.916	I$_{(86/22)}$	M$_{(78/14)}$	M	3		
	208	0.947	L$_{(100/0)}$	V$_{(80/0)}$	L	3		
NS5	20	0.916	K$_{(68/24)}$	R$_{(76/32)}$	K	3		
	31	0.926	I$_{(90/18)}$	V$_{(82/10)}$	V	3		
	44	0.919	R$_{(96/7)}$	K$_{(93/3)}$	K	3		
	113	0.916	K$_{(84/7)}$	R$_{(93/16)}$	R	3		
	162	0.958	K$_{(75/22)}$	R$_{(78/25)}$	R	3		
	**226**	1.000	T$_{(100/0)}$	S$_{(100/0)}$	**T**	3		
	260	0.920	V$_{(82/22)}$	T$_{(78/14)}$	V	3		
	**290**	1.000	E$_{(99.6/0)}$	D$_{(100/0.4)}$	D	3		
	404	0.958	K$_{(78/22)}$	R$_{(78/22)}$	R	3		
	590	0.958	I$_{(80/22)}$	V$_{(78/20)}$	V	3		
	696	0.950	H$_{(78/22)}$	P$_{(78/22)}$	P	3		
	854	0.947	K$_{(96/0)}$	R$_{(100/4)}$	G	3		
	872	0.979	K$_{(96/4)}$	R$_{(96/4)}$	K/R	1/2		
	890	0.960	D$_{(99/0)}$	E$_{(100/0)}$	D	3		

Blue color – encephalitic form
Red color – hemorrhagic form
Green color – unique LGTV aa residues
* - absolutely specific positions are bolded
