# Supplementary figures and images for: Genomic Determinants Potentially Associated with Clinical Manifestations of Human-Pathogenic Tick-Borne Flaviviruses
Source: Int J Mol Sci. 2022 Nov 2;23(21):13404. doi: 10.3390/ijms232113404 (PMC9658301; doi:10.3390/ijms232113404)

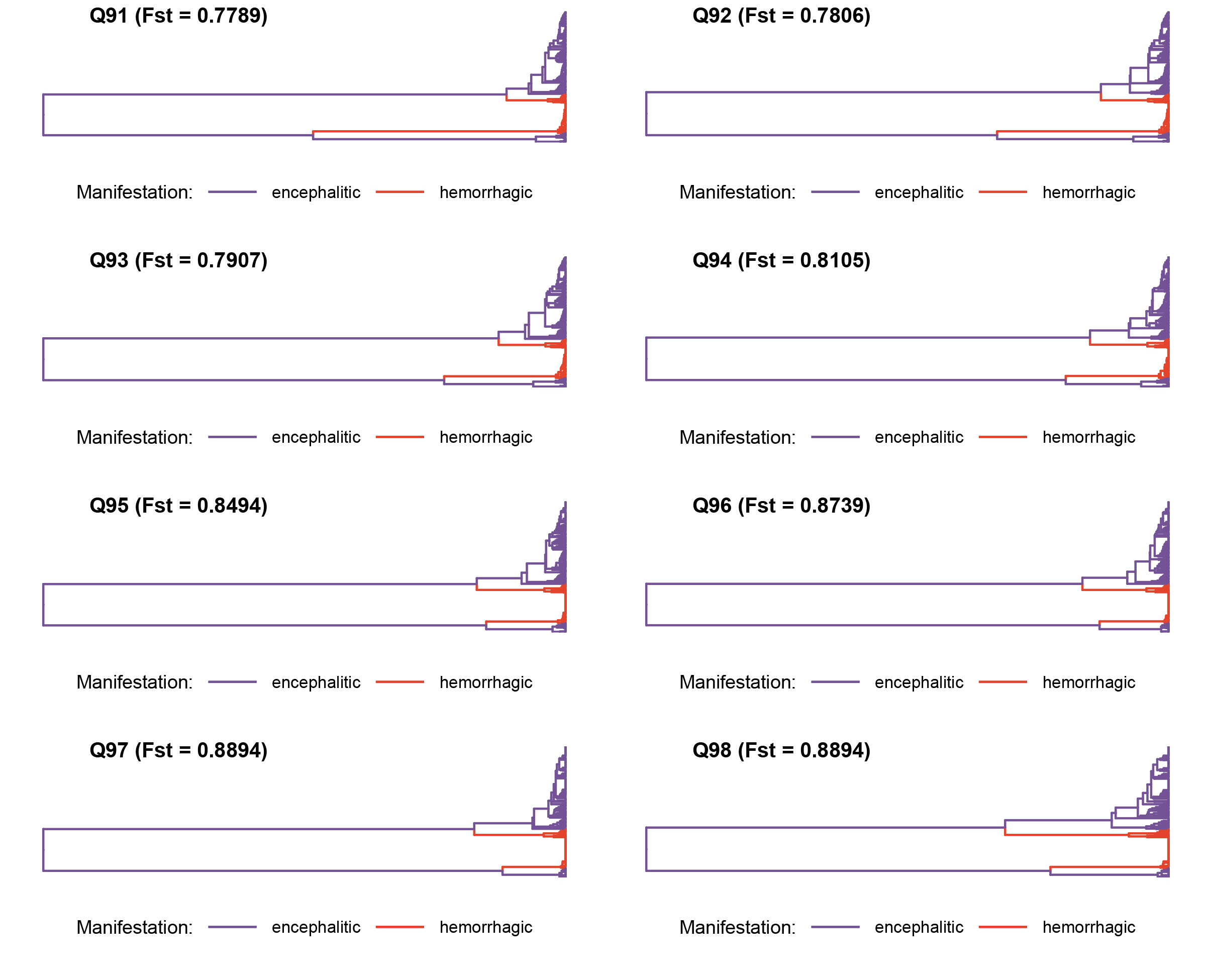

Supplement: Supplementary file 1 [file ijms-23-13404-s001.zip › Figure S1.tif]
